# Effects of compound yeast culture on growth performance, antioxidant function and inflammatory factors of Hu sheep

**DOI:** 10.3389/fvets.2025.1674231

**Published:** 2025-09-23

**Authors:** Shixiong Liu, Jiabin Ma, Lan Yang, Hui Chen, Xueqiang Li, Rui Du, Chen Xue, Dacheng Liu

**Affiliations:** ^1^College of Veterinary Medicine, Inner Mongolia Agricultural University, Hohhot, China; ^2^Key Laboratory of Clinical Diagnosis and Treatment Techniques for Animal Disease, Ministry of Agriculture, Hohhot, China

**Keywords:** antioxidant function, compound yeast culture, Hu sheep, inflammatory factors, production performance

## Abstract

**Introduction:**

The purpose of this study was to investigate the effects of compound yeast culture on production performance, antioxidant function, and inflammatory factors in Hu sheep.

**Methods:**

A total of 180 45-day-old healthy Hu sheep were randomly divided into two groups. The control group was fed a basal diet, and the experimental group was supplemented with 50 g/kg compound yeast culture during the conservation period (1–30 days) and 60 g/kg during the fattening period (31–117 days). The experiment lasted 124 days, with a pre-feeding period of 7 days and a formal period of 117 days. Daily feed intake was recorded, and the animals were weighed before morning feeding on days 1, 36, 67, 97, and 117 of the experiment and slaughtered on the 117th day. At the same time, non-anticoagulant blood was collected before morning feeding on days 1, 30, 60, and 90, and serum antioxidant indices and serum inflammatory factors were measured.

**Results:**

The results showed that the average daily gain of Hu sheep in the experimental group increased by 38 g compared to that in the control group, which increased by 13.4% (*p* < 0.05). The average daily feed intake of the experimental group increased by 80 g, an increase of 5% (*p* < 0.05). The feed-to-weight ratio in the experimental group decreased by 8.3% (*p* < 0.05). Compared with the control group, the economic profit of the experimental group increased by 130.27 yuan, an increase of 34.6% (*p* < 0.01). The serum total antioxidant capacity (T-AOC) content and superoxide dismutase activity (SOD) activity of Hu sheep in the experimental group significantly increased (*p* < 0.05). The malondialdehyde (MDA) content decreased significantly (*p* < 0.01). The contents of inflammatory factors TNF-*α*, IL-6, IL-4 and IL-10 in the serum of Hu sheep in the experimental group were significantly increased (*p* < 0.05).

**Discussion:**

The above results showed that compound yeast culture significantly increased Hu sheep’s feed intake, daily gain, and economic benefit, and significantly reduced the feed-to-weight ratio, improved the antioxidant capacity, improved the immune function, and alleviated the effect of stress on Hu sheep.

## Highlights

- Improved productivity: yeast culture boosted feed intake, daily gain, and feed efficiency.
- Higher profitability: economic returns increased by over 30%.
- Better health outcomes: antioxidant capacity and immune resilience were enhanced.
- Wider potential: yeast culture shows promise for other ruminant production systems.

## Introduction

In the breeding process, free radicals are often increased due to long-distance transportation, environmental changes, and poor diet (1–3). A large number of free radicals are produced, leading to the accumulation of free radicals, causing damage to the animal body, destroying the body’s proteins, nucleic acids, and biofilm system, and causing oxidative stress and various inflammatory reactions (4–6). Recent studies have shown that yeast culture has positive effects on animal production performance and antioxidant capacity (7–10). Studies have shown that the addition of yeast culture to diet improves the growth and slaughter performance of mutton sheep (11), increases animal milk production (12), reduces somatic cell count (13), and improves rumen microflora (14, 15). Feeding the yeast culture to Simmental cattle not only enhanced the growth performance but also increased the activity of glutathione peroxidase (GSH-Px) and total antioxidant capacity (T-AOC) in serum, reduced the content of malondialdehyde (MDA), and enhanced the antioxidant function of the body. Adding yeast culture to the diet of dairy cows can increase the (SOD) and total antioxidant capacity of lactating cows (16).

Compound yeast culture is a microecological preparation developed in our laboratory based on the physiological characteristics of ruminants. In a previous study, it was found that compound yeast culture can significantly improve the immunity of small-tailed Han sheep and significantly improve the weight gain, slaughter rate, and carcass weight of mutton sheep (17). A study on pigs, revealed that feeding compound yeast culture could improve production performance and reduce the number of stillbirths and constipation in sows (18). In a study on beef cattle, a compound yeast culture was found to improve immunity and provide economic benefits in animals (16). This study further explored the effects of compound yeast culture on growth performance, antioxidant capacity, and inflammatory factors in Hu sheep and provided a theoretical basis for the application of compound yeast culture in Hu sheep breeding.

## Materials and methods

### Preparation of yeast culture

The compound yeast culture was fermented with 15% wheat bran, 12% corn bran, 16% corn meal, 10% rice bran meal, 8% DDGS, 27% corn germ meal, and 12% soybean meal. The fermentation strains, *Saccharomyces cerevisiae* XR4 and *Kluyveromyces marxianus* BC, were isolated by our research group with independent intellectual property rights. The two strains were mixed in a ratio of 1:1 to make a fermentation broth (1.5 × 10^8^ cfu/g). Inoculation was performed at a dose of 10% of the solid substrate mass, and a certain amount of water was added to make the initial water content of the fermentation substrate 38–40%. Solid-state stacking fermentation was performed in a fermentation workshop with a stacking height of 60–65 cm. The temperature of the material was recorded every 3 h during fermentation. When the fermentation time reached 24 h and the temperature reached more than 40°C, the material was turned over once. After the material was turned over, stacking fermentation continued. The entire fermentation process lasted for 72 h. After fermentation, low-temperature drying and crushing bagging were performed, and the preparation of the composite yeast culture was completed. Nutritional indicators and amino acid compositions are shown in Tables 1, 2, respectively.

**Table 1:** Nutritional level of compound yeast culture.

Item	Compound yeast culture
Dry matter, %	91.79
Crude protein, %	21.71
Crude fat, %	3.56
Coarse ash, %	6.01
Crude fiber, %	9.10
Acid-soluble protein, %	5.82
Live yeast cells, CFU/g	5.1 × 10^4^
Lactic acid, mmol/gprot	1.49

**Table 2:** Amino acid content in compound yeast culture.

Item	Compound yeast culture
The sum of 16 amino acids, %	19.42
aspartate, %	1.77
threonine, %	0.78
serine, %	0.93
glutamate, %	3.41
proline, %	1.00
glycine, %	0.94
alanine, %	1.20
valine, %	1.01
isoleucine, %	0.65
leucine, %	1.70
tyrosine, %	0.57
phenylalanine, %	0.85
tryptophan, %	0.00
histidine, %	0.61
lysine, %	0.88
Arginine, %	1.08

### Animals and diet

The experiment was conducted at the Hu sheep breeding base in Qingyang City, Gansu Province, China. A total of 180, 45-day-old weaned healthy lambs were randomly divided into two groups (*n* = 90 per group). The trial period was 124 days, including 7 days of prefeeding and 117 days of the trial period. During the prefeeding period, the lambs were disinfected, epidemic prevention, earmarked, and dewormed, according to the routine procedures of the sheep farm. Feed at 08:00 and 18:00 every day, with free access to water.

The nutritional requirements for different growth stages vary. The fattening process was divided into a conservation period (1–30 days of age) and a fattening period (31–117 days of age). The control group was fed the basic diet of the sheep farm, and the experimental group was fed the basic diet. Compound yeast culture was added at 50 g/kg during the conservation period and at 60 g/kg during the fattening period. The basal diet was formulated according to mutton sheep feeding standards’ (NY/T 816-2004). The energy and protein levels in each group were the same, and the feed composition is shown in Table 3.

**Table 3:** Ingredient composition and nutrient levels of diets (dry matter basis).

Items	Pre-test	Late test
Ingredients		
Corn	26	32.3
Soybean meal	5	6
Germ meal	10	8
Cottonwood	6	7
Wine trough protein	4	4
Stone powder	0.7	0.7
5% premix^a^	3.8	1.5
Nh_4_Cl	0.5	0.5
Tonco	5	5
NaCl	0.5	0.5
CaHPO_4_	1.1	1.0
Compound yeast cultures	5	6
Alfalfa meal	21.5	22.5
Peanut shell	5	6
Oil bran	5	0
Total	100	100
Nutrients^b^		
DE (MJ/kg)^b^	10.87	12.22
DM/%	89.82	88.64
CP/%	16.31	14.53
NDF/%	29.69	23.03
ADF/%	17.76	13.01
Ca/%	1.41	0.99
P/%	0.58	0.44

a The premix provided the following per kg: VB_12_ 0.7 mg, VA 350000 IU, VB_2_ 188 mg, VB_3_ 750 mg, VB_5_ 500 mg, VB_6_ 2 mg, VB_7_ 3.7 mg, VB_9_ 38 mg, VD_3_ 93,750 IU, VE 938 mg, VK_3_ 63 mg, Se 18 mg, Zn 3,000 mg, I 23 mg, Co30 mg, Mn 2,500 mg. Fe3240 mg. Cu 500 mg.

b DE was a calculated value, whereas other nutrient levels were measured values.

### Determination method of dietary nutrient levels

The dry matter (DM) content was determined according to the method of GB/T 6435-2014, crude protein (CP) content was determined according to the method of GB/T 6432-2018, crude fat (EE) content was determined according to the method of GB/T 6433-2006, crude ash (Ash) content was determined according to the GB/T 6438-2007 method, and crude fiber (CF) content was determined according to the method of GB/T 6434-2006. The neutral detergent fiber (NDF) content was determined according to GB/T 20806-2022 method, content of acid detergent fiber (ADF) content was determined according to the NY/T 1459-2007 method, acid soluble protein (ASCP) content was determined according to the NY/T 3801-2020 method, number of live yeast was determined according to the GB method 7300.501-2021, and amino acid content was determined according to the GB/T 18246-2019 method. Calcium (Ca) content was determined according to the GB/T 6436-2018 method, phosphorus (P) content was determined according to the GB/T 6437-2018 method, and lactic acid was determined using a lactic acid detection kit (no. G4308, Wuhan Sevier Biotechnology Co. Ltd., Wuhan, China). Digestible energy (DE) was calculated using the Refs3000 software.

### Sample collection and detection

#### Determination of growth performance indexes

After the start of the experiment, leftovers were accurately weighed daily and the daily feed intake of the animals was calculated. The average daily gain and feed-to-weight ratio were calculated by weighing the sheep on days 1, 36, 67, 97, and 117, weighed on an empty stomach on the last day of the trial period and slaughtered every other day. Carcass weight was measured on the day of slaughter, and the slaughter rate and economic benefits were calculated. The relevant formulae are as follows:


$$\begin{array}{c} \text{Average daily gain}\;(g/d)=(\text{final weight}-\text{initial weight})\\ /(\text{test days}\times \text{number of sheep}) \end{array}$$

$$\begin{array}{c} \text{Average daily feed intake}=\text{total feed intake}\\ /(\text{test days}\times \text{number of sheep}) \end{array}$$

Feed to gain ratio=average daily feed intake/average daily gain

$$\begin{array}{l} \text{Carcass weight}\;(\mathrm{kg})=\text{live weight before slaughter}\\ -\{\text{weight of head},\text{hoof},\text{skin},\text{tail and viscera}\;(\text{kidney})\} \end{array}$$

Slaughter rate(%)=100%×carcass weight/pre−slaughter weight

Feed cost(yuan/only)=feed intake(kg)×feed unit price(yuan)

$$\begin{array}{c} \text{Meat production benefit}\;(\text{yuan}/\text{only})=\text{carcass weight}\;(\mathrm{kg})\\ \times \text{mutton price}\;(\text{yuan}) \end{array}$$

Production profit(yuan/only=meat production benefit−feed cost)

#### Serum sample collection

Fifteen sheep were randomly selected from each group, and blood was collected from the jugular vein before morning feeding on days 1, 30, 60, and 90. The serum was placed at 4°C for 24 h, and the isolated serum was stored in a sterile non-enzymatic cryopreservation tube. It was stored at −20°C for the detection of antioxidant serum indices and inflammatory factors.

#### Analysis of serum indexes of immune function

The levels of cytokines interleukin-1β (IL-1β), IL-2, IL-4, IL-6, IL-10, and tumor necrosis factor-*α* (TNF-α) in serum were detected by enzyme-linked immunosorbent assay (ELISA) kit. All ELISA kits were purchased from Quanzhou Ruixin Biotechnology Co. Ltd. Detection was performed in strict accordance with manufacturer’s instructions. The microplate reader used in this experiment was an Agilent Technologies 800TS-SN microplate reader (United States). Information on the kit is shown in Supplementary Table 1.

#### Analysis of serum indexes of antioxidant capacity

MDA content was detected using the thiobarbituric acid method, superoxide dismutase (SOD) activity was detected by the xanthine oxidase method, catalase (CAT) activity was detected by the ammonium molybdate method, glutathione peroxidase (GSH-Px) activity was detected by colorimetry, and total antioxidant capacity (T-AOC) was detected by the Fe^3+^ reduction method. Information on the kit is shown in Supplementary Table 2.

### Data analysis

Excel 2020 was used to process and calculate experimental data for each group. Statistical software (SAS 9.0) was used to analyze the data using one-way analysis of variance. Multiple comparisons were made using the least significant difference method (*p* < 0.05, significant *p* < 0.01, extremely significant; *p* > 0.05, not significant).

## Results

### Effects of compound yeast culture on production performance of Hu sheep

As shown in Table 4, on the 67th day of the experiment, the body weight, average daily feed intake, daily gain, and feed-to-weight ratio of the experimental group were not significantly different from those of the control group (*p* > 0.05). During the period of 68–97 days, the average daily gain of Hu sheep in the experimental group increased by 106.7 g compared to that in the control group, which increased by 43.5% (*p* < 0.05). The average daily feed intake of the experimental group was 90 g higher than that of the control group, an increase of 4.8% (*p* < 0.05). On day 97 of the experiment, the weight gain of Hu sheep in the experimental group was 3.6 kg, which was 8.1% higher than that in the control group (*p* < 0.01). On experiment day 117, the average weight of the experimental group was 53.70 kg, which was 4.14 kg higher than that of the control group, an increase of 8.3% (*p* < 0.01); the average daily gain of the experimental group was 320.27 g, which was 38.04 g higher than that of the control group and increased by 13.4% (*p* < 0.05). Feed intake of the experimental group was 80 g higher than that of the control group, an increase of 5% (*p* < 0.05). The feed-to-weight ratio in the experimental group decreased by 8.3% (*p* < 0.05). The above results show that the growth performance of Hu sheep could be improved by feeding them a compound yeast culture.

**Table 4:** Effects of compound yeast culture on production performance of Hu sheep.

Items	Time	Control group	Trial group	*p*-value	Relative change (%)
BW/kg	Day 1	16.31 ± 3.86	16.22 ± 3.88	0.280	−0.55
	Day 36	27.48 ± 4.96	27.68 ± 5.51	0.685	0.73
	Day 67	36.89 ± 6.55	37.41 ± 6.92	0.491	1.41
	Day 97	44.36 ± 6.63^b^	47.96 ± 8.13^a^	<0.001	8.12
	Day 117	49.56 ± 8.12^b^	53.70 ± 8.86^a^	<0.001	8.35
ADG/(g/d)	1 to 36 days	307.25 ± 15.97	324.70 ± 22.65	0.187	5.68
	37 to 67 days	300.33 ± 30.06	309.27 ± 26.57	0.673	2.98
	68 to 97 days	245.00 ± 75.03^b^	351.67 ± 41.67^a^	<0.05	43.54
	98 to 117 days	252.13 ± 90.77	280.17 ± 14.10	0.602	11.12
	1 to 117 days	282.23 ± 19.09^b^	320.27 ± 16.23^a^	<0.05	13.48
ADF (kg/d)	1 to 36 days	1.14 ± 0.10	1.18 ± 0.12	0.054	3.51
	37 to 67 days	1.69 ± 0.17	1.76 ± 0.19	0.078	4.14
	68 to 97 days	1.85 ± 0.16^b^	1.94 ± 0.15^a^	<0.05	4.86
	98 to 117 days	1.95 ± 0.17	2.06 ± 0.09	0.168	5.64
	1 to 117 days	1.61 ± 0.15^b^	1.69 ± 0.14^a^	<0.05	4.97
F/G	1 to 36 days	3.83 ± 0.33	3.67 ± 0.19	0.288	−4.18
	37 to 67 days	5.66 ± 0.31	5.68 ± 0.39	0.951	0.35
	68 to 97 days	7.67 ± 1.20	5.52 ± 0.55	0.075	−28.03
	98 to 117 days	8.47 ± 3.07	7.38 ± 0.42	0.591	−12.87
	1 to 117 days	5.70 ± 0.17^a^	5.26 ± 0.18^b^	<0.05	−7.72

In the same row, values with no letters or the same letter superscripts indicate no significant differences (*p* > 0.05), whereas different superscript letters indicate significant differences (*p* < 0.05), *n* = 90. BW, body weight; ADG, average daily gain; ADF, average daily feed intake; F/G, ADF/ADG.

### Effect of compound yeast culture on economic benefit of Hu sheep

In this experiment, the conservation period (1–30 d) based diet was 3,800 yuan/ton, containing a compound bacterial culture feed of 3,600 yuan/ton. The fattening period (31 to 117 d) included a basal diet of 3,200 yuan/ton, compound bacterial culture feed of 3,000 yuan/ton, corn of 3,000 yuan/ton, and Hu wool weight price of 30 yuan/kg. The experimental group invested 2,216 yuan more than the control group. Cycle additional investment of 50 yuan (water, electricity, and labor costs). As shown in Table 5, the production profit of the experimental group increased by 130.27 yuan, which is an increase of 34.6% (*p* < 0.01).

**Table 5:** Effect of compound yeast culture on economic benefit of Hu sheep.

Items	Control group	Trial group	*p*-value	Relative change (%)
Feed costs of days (yuan/only)	613.65 ± 56.88	616.84 ± 53.50	0.749	0.52
Meat benefits (yuan/only)	990.65 ± 66.98	1124.11 ± 57.04	0.016	13.47
Production profits (yuan/only)	377.00 ± 11.60	507.27 ± 6.52	0.004	34.55
Profits comparison (yuan/only)	0	130.27		

In the same row, values with no letters or the same letter superscripts indicate no significant differences (*p* > 0.05), whereas different superscript letters indicate significant differences (*p* < 0.05), *n* = 90.

### Effects of compound yeast culture on slaughter performance of Hu sheep

As shown in Table 6, the slaughter indexes of the experimental group were higher than those of the control group; however, the difference was not significant.

**Table 6:** Effects of compound yeast culture on slaughter performance of Hu sheep.

Items	Control group	Trial group	*p*-value	Relative change (%)
pH	6.45 ± 0.26	6.51 ± 0.37	0.449	0.93
LWBS (kg)	44.75 ± 5.49	47.25 ± 4.59	0.191	5.59
CW (kg)	23.22 ± 3.33	24.84 ± 2.60	0.160	6.98
Dressing percentage (%)	51.75 ± 1.72	52.55 ± 1.44	0.230	1.55

LWBS = live weight before slaughtering, CW = carcass weight, Mean ± SD, the same row, values with no letter or the same letter superscripts mean no significant difference (*p* > 0.05), while different letter superscripts mean significant difference (*p* < 0.05), *n* = 90.

### Effect of compound yeast culture on antioxidant capacity index of Hu sheep

**Figure 1:**
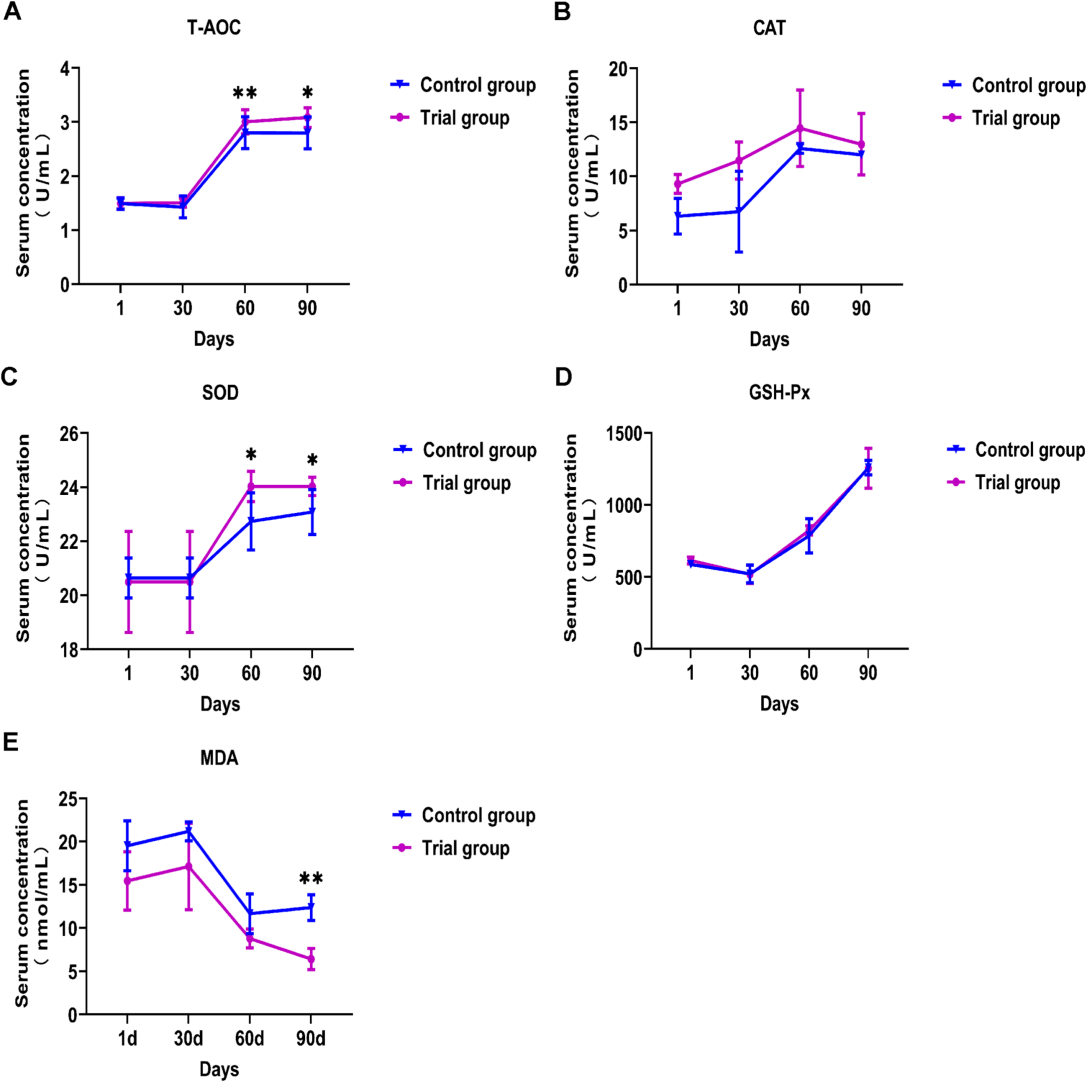
Effects of compound bacteria culture on serum antioxidant indexes of Hu sheep. **(A)** Effects on the serum T-AOC activity. **(B)** Effects on the serum CAT activity. **(C)** Effects on the serum SOD activity. **(D)** Effects on the serum GSH-Px activity. **(E)** Effects on the serum MDA concentration. SOD = Superoxide dismutase, T-AOC = Total antioxidant capacity, GSH-Px = Glutathione peroxidase, CAT = Catalase, MDA = Malondialdehyde. Mean ± SD; * (*p* < 0.05) vs. control; ** (*p* < 0.01) vs. control. T-test, *n* = 5.

As shown in Figure 1, there were no significant differences in the five indicators between the experimental and control groups during the first month of the experiment. When the experiment was performed for 60 d, SOD activity in the serum of Hu sheep in the experimental group was significantly higher than that in the control group (*p* < 0.05), and serum T-AOC in the experimental group was significantly increased (*p* < 0.01). On day 90, the SOD activity and T-AOC in the serum of Hu sheep in the experimental group were significantly higher than those in the control group (*p* < 0.05), and the MDA content in the experimental group was significantly lower than that in the control group (*p* < 0.01). Throughout the experiment, the serum SOD activity and T-AOC of the experimental group increased significantly, whereas the MDA content decreased significantly. There was no significant change in serum CAT and GSH-Px activity in the experimental group compared to the control group. These results showed that feeding compound yeast culture could improve the antioxidant capacity of Hu sheep.

### Effects of compound yeast culture on the content of inflammatory factors in Hu sheep

**Figure 2:**
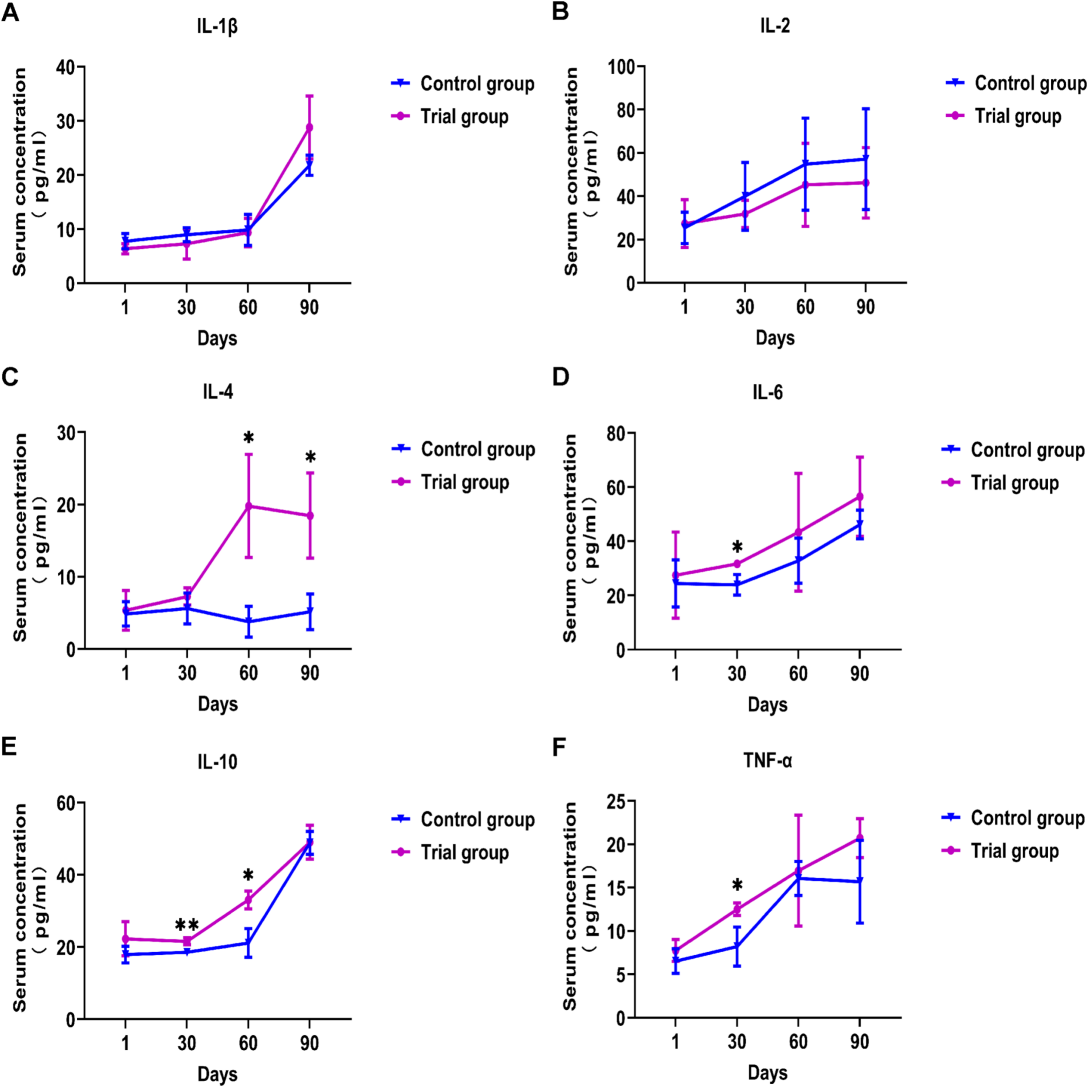
Effects of compound yeast culture on the content of inflammatory factors in Hu sheep. **(A)** Effects on the serum IL-1β concentration. **(B)** Effects on the serum IL-2 concentration. **(C)** Effects on the serum IL-4 concentration. **(D)** Effects on the serum IL-6 concentration. **(E)** Effects on the serum IL-10 concentration. **(F)** Effects on the serum TNF-α concentration. IL-1β = Interleukin-1β, IL-2 = Interleukin-2, IL-4 = Interleukin-4, IL-6 = Interleukin-6, IL-10 = Interleukin-10, TNF-α = Tumor necrosis factor-α. Mean ± SD; * (*p* < 0.05) vs. control; ** (*p* < 0.01) vs. control. T-test, *n* = 5.

As shown in Figure 2, there was no significant difference in serum inflammatory factors between the experimental and control groups at the beginning of the experiment. The serum levels of TNF-*α*, IL-6, and IL-10 were significantly increased at 1 month (*p* < 0.05). On day 60, the serum levels of IL-4 and IL-10 in the experimental group were significantly higher than those in the control group (*p* < 0.05). On day 90, the serum IL-4 level in the experimental group was significantly higher than that in the control group (*p* < 0.05). Throughout the test period, the serum TNF-α, IL-6, IL-4, and IL-10 contents of the experimental group were significantly increased, and the differences of IL-1β and IL-2 were not significant. These results suggest that supplementation with compound yeast culture supplementation modulates immune-related cytokines in Hu sheep.

## Discussion

### Effects of compound yeast culture on growth performance of Hu sheep

This study was conducted at the Hu sheep breeding base in Qingyang City, Gansu Province. Hu sheep have the advantages of requiring little exercise and growing rapidly, but they also have poor disease resistance and slow digestion (19, 20). In this study, the average daily feed intake, average daily gain, and feed-to-weight ratio of Hu sheep in the experimental group significantly increased after feeding with the compound yeast culture. The digestive and absorptive capacities of the gastrointestinal tract determine the feed intake of Hu sheep. The key to fattening Hu sheep is the nutritional content, palatability, and digestibility of the feed itself (21–23). In this study, the feed intake of Hu sheep in the experimental group was significantly higher than that in the control group. Compound yeast cultures are rich in various nutritionally active substances and probiotics (24, 25). Under the synergistic effect of nutritionally active substances and probiotics, the digestibility of crude protein and dry matter improved (26), and the decomposition, digestion, and absorption of nutrients in Hu sheep feed were enhanced. It promotes the growth of beneficial bacteria in the gastrointestinal tract of Hu sheep, strengthens the digestive and absorptive functions of the intestinal tract, and improves the growth performance of Hu sheep (27). In contrast, compound yeast cultures have a unique fermented acid aroma (28), which can stimulate the appetite of animals and increase feed intake in Hu sheep. Compound yeast culture also increased the feed conversion rate and feed intake of Hu sheep. This study also found that the economic benefit of Hu sheep after feeding with a compound yeast culture was 34.6% higher than that of the control group, which also proved that adding a compound bacterial culture to feed could reduce the feeding cost of the farm and help shorten the growth cycle of Hu sheep. Various studies have shown differences in whether yeast culture can significantly improve slaughter performance. Studies have shown that adding yeast culture to sheep diets has been found to significantly increase the slaughter rate of animals (29–31). Some studies have indicated that yeast culture has no effect on the slaughter performance of mutton sheep (32). Additionally, this demonstrates the results of this experiment. In this experiment, the slaughter data were higher than those of the control group; however, the difference was not significant. This shows that the slaughter performance of Hu sheep fed a compound bacterial culture can be improved.

### Effects of compound yeast culture on serum antioxidant indexes of Hu sheep

Under normal circumstances, the production and scavenging of free radicals in the body are in a state of dynamic balance (33, 34). This experiment has been conducted over 117 days in the Hu sheep feeding test. In this process, Hu sheep often encounter the situation of the Hu sheep turning circle, sudden change in sheep circle temperature, excessive noise from cleaning vehicles, scattering vehicles, etc., which will lead to a strong stress response in Hu sheep. A study by Chen et al. showed that feeding polysaccharides could increase the T-AOC and SOD activity in the serum of mutton sheep (35) and enhance the antioxidant capacity of animals. T-AOC is an overall evaluation index of antioxidant levels in the body (36, 37). SOD represents the ability of the animal body to scavenge oxygen free radicals (38–40). Functional additives reduce oxidative stress by reducing the formation of free radicals. In this study, the serum T-AOC and SOD activity of the experimental group significantly increased, indicating that feeding the compound yeast culture can improve the antioxidant capacity of Hu sheep and reduce damage caused by oxidative stress. In this experiment, Hu sheep experienced a sudden change in temperature and continuous light rain for many days. The results showed that the Hu sheep in the experimental group did not show an obvious stress response, and their feed intake was normal. The control group not only showed a significant decrease in feed intake, but also death, which further confirmed that the compound yeast culture could improve the anti-oxidative stress ability of Hu sheep. MDA is the best index of oxidative stress and can be used to measure the degree of oxidative damage (41–43). On the 90th day of the experiment, the serum MDA content of the experimental group was significantly reduced, which proved that the addition of compound yeast culture to the diet improved the antioxidant capacity of Hu sheep. Previous studies have shown that supplementation with yeast cultures can improve the antioxidant capacity of animals (44). These findings correspond with the results of our experiment. After feeding the compound yeast culture, it was beneficial for Hu sheep to scavenge free radicals produced during the metabolic process, reduce the damage caused by oxygen free radicals to the body, and improve the antioxidant capacity of Hu sheep. Lu et al. reported that yeast culture can improve the antioxidant capacity of Sheep Livers and reached a similar conclusion to this experiment (45). There was no significant change in serum GSH-Px activity, which differs from the results of previous studies (36). This may be due to the excessive consumption of concentrate, resulting in nutritional metabolic oxidative stress in Hu sheep.

### Effect of compound yeast culture on the content of serum inflammatory factors in Hu sheep

Although the cytokine content in animal serum is low, it plays an important role in the body’s defense against diseases (46). It mediates the interactions between cells, participates in inflammatory responses, and regulates immune responses. Therefore, the health status of Hu sheep can be determined using cytokine detection. Under normal circumstances, the body’s pro-inflammatory and anti-inflammatory factors are in dynamic balance (47). When the body has a disease or changes in the external environment, it will lead to an increase in pro-inflammatory factors in the body. An appropriate amount of pro-inflammatory factors activates the body’s immune function, while excessive pro-inflammatory cytokines lead to an inflammatory response in the body. TNF-*α* can enhance infection resistance by activating neutrophils and platelets, enhancing the killing ability of macrophages/NK cells, and stimulating the immune system. IL-6 promotes the proliferation and differentiation of B-lymphocytes to produce antibodies (48). TNF-α and IL-6 are involved in the regulation of immune activity, thereby enhancing animal immunity (49). Both IL-1β and IL-2 are pro-inflammatory factors, and the level of IL-2 cytokines will increase after stress (50). IL-1β has a strong pro-inflammatory ability in neutrophil infiltration and is a strong pro-inflammatory factor (51). During the experiment, the serum levels of TNF-α, IL-6, IL-4, and IL-10 in the experimental group were significantly increased. On the one hand, an appropriate increase in cytokine content can help the body promote the immune response (52); on the other hand, the content of IL-4 and IL-10 increases significantly in harsh environments, which can inhibit the occurrence of inflammation and tissue damage and alleviate the damage caused by stress in animals (53–55). The serum IL-1β and IL-2 in the experimental group did not change significantly compared with the control group. It may be because the increase of IL-4 and IL-10 inhibited pro-inflammatory factors such as IL-1β. IL-4 can inhibit the activity of pro-inflammatory cytokines and IL-10 can inhibit lymphocytes to reduce inflammation (56, 57). In this experiment, the content of serum IL-4 and IL-10 increased, combined with the determination of antioxidant indices, which proved that the immune ability of Hu sheep was enhanced by feeding with the compound yeast culture.

## Conclusion

This experiment was conducted to study the effects of a compound yeast culture on the growth performance, immune function, and antioxidant capacity of Hu sheep. The results showed that the addition of the compound yeast culture to the diet improved their growth performance, immune function, and antioxidant capacity. Therefore, compound yeast culture is considered a potential antibiotic substitute that can be used as an animal feed additive. These data provide theoretical support for the development of mutton sheep breeding programs.

## Data Availability

The original contributions presented in the study are publicly available at: https://figshare.com/articles/dataset/dx_doi_org_10_6084_m9_figshare_6025748/6025748.
