# Supplementary material for: Effects of compound yeast culture on growth performance, antioxidant function and inflammatory factors of Hu sheep
Source: Front Vet Sci. 2025 Sep 23;12:1674231. doi: 10.3389/fvets.2025.1674231 (PMC12502084; doi:10.3389/fvets.2025.1674231)
Supplement: Supplementary file 1 [file Table_1.docx]

## Supporting Information

## Table：

Table S1 Serum immune index kits and their product numbers.

| Items | Kit names | Product numbers |
| --- | --- | --- |
| IL-1β | [Interleukin -1β Assay Kit](http://www.njjcbio.com/products.asp?id=557) | RX800386SH |
| IL-2 | [Interleukin -2 Assay Kit](http://www.njjcbio.com/products.asp?id=3589) | RX800384SH |
| IL-4 | [Interleukin -4 Assay Kit](http://www.njjcbio.com/products.asp?id=560) | RX800383SH |
| IL-6 | [Interleukin -6](http://www.njjcbio.com/products.asp?id=3395) | RX800382SH |
| IL-10 | [Interleukin -10 Assay Kit](http://www.njjcbio.com/products.asp?id=564) | RX800292SH |
| TNF-α | [Tumor Necrosis Factor-α Assay Kit](http://www.njjcbio.com/products.asp?id=3519) | RX800330SH |

Table S2 Serum antioxidant index kits and their product numbers.

| Items | Kit names | Product numbers |
| --- | --- | --- |
| MDA | [Malondialdehyde (MDA) assay kit (TBA method)](http://www.njjcbio.com/products.asp?id=287) | [A003-1-2](http://www.njjcbio.com/products.asp?id=287) |
| SOD | [Total Superoxide Dismutase (T-SOD) assay kit (Hydroxylamine method)](http://www.njjcbio.com/products.asp?id=284) | [A001-1-2](http://www.njjcbio.com/products.asp?id=284) |
| CAT | [CATalase (CAT) assay kit (Visible light)](http://www.njjcbio.com/products.asp?id=299) | [A007-1-1](http://www.njjcbio.com/products.asp?id=299) |
| GSH-Px | [Glutathione Peroxidase (GSH-Px) assay kit (Colorimetric method)](http://www.njjcbio.com/products.asp?id=297) | [A005-1-2](http://www.njjcbio.com/products.asp?id=297) |
| T-AOC | [Total antioxidant capacity assay kit](http://www.njjcbio.com/products.asp?id=313) | [A015-1-2](http://www.njjcbio.com/products.asp?id=313) |
